# Somatotropic Axis, Pace of Life and Aging

**DOI:** 10.3389/fendo.2022.916139

**Published:** 2022-07-14

**Authors:** Andrzej Bartke

**Affiliations:** Department of Internal Medicine, Southern Illinois University School of Medicine, Springfield, IL, United States

**Keywords:** lifespan, longevity, growth hormone, somatotropic axis, mutant mice, pace of life, aging

## Abstract

Mice with genetic growth hormone (GH) deficiency or GH resistance live much longer than their normal siblings maintained under identical conditions with unlimited access to food. Extended longevity of these mutants is associated with extension of their healthspan (period of life free of disability and disease) and with delayed and/or slower aging. Importantly, GH and GH-related traits have been linked to the regulation of aging and longevity also in mice that have not been genetically altered and in other mammalian species including humans. Avai+lable evidence indicates that the impact of suppressed GH signaling on aging is mediated by multiple interacting mechanisms and involves trade-offs among growth, reproduction, and longevity. Life history traits of long-lived GH-related mutants include slow postnatal growth, delayed sexual maturation, and reduced fecundity (smaller litter size and increased intervals between the litters). These traits are consistent with a slower pace-of-life, a well-documented characteristic of species of wild animals that are long-lived in their natural environment. Apparently, slower pace-of-life (or at least some of its features) is associated with extended longevity both within and between species. This association is unexpected and may appear counterintuitive, because the relationships between adult body size (a GH-dependent trait) and longevity within and between species are opposite rather than similar. Studies of energy metabolism and nutrient-dependent signaling pathways at different stages of the life course will be needed to elucidate mechanisms of these relationships.

## Introduction

### Differences of Longevity Between and Within Species

**Figure 1:**
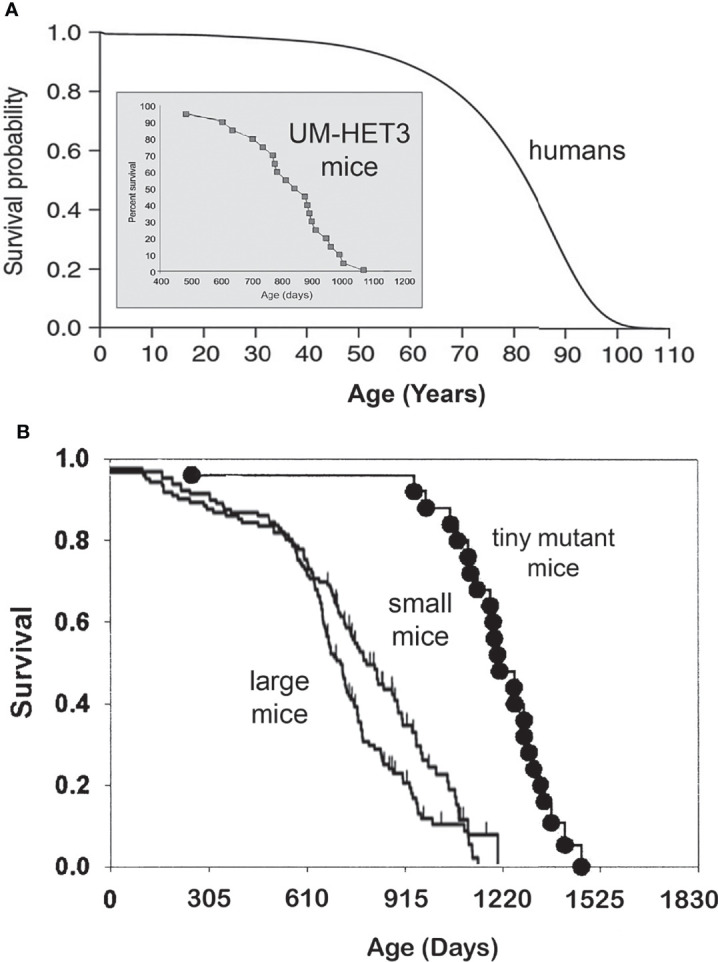
**(A)** Survival curves of a short-lived mammal, the mouse, and a long-living mammal, the human. Please note different time scales, days vs. years. Human survival curve based on Arias E. United States life tables, 2010 (1), and mouse longevity based on Miller et al. (2) and Flurkey et al. (3). **(B)** In a genetically heterogeneous population of normal (wild type) mice, smaller individuals live longer. Mice with hereditary dwarfism live much longer than WT mice (4, 5).

Longevity, measured by average, median, or maximal lifespan, varies greatly among different species. The range of this variability is enormous, from hours and days to hundreds of years. Among mammals, maximal longevity ranges from two to three years in some small rodents, to well over a hundred years in some species of whales. In general, larger animals live longer, such as the example of mice and humans (**Figure 1A**), but there are many exceptions. For example, primates, including monkeys, great apes, and humans, live longer than carnivores or ungulates of the same body size. Moreover, rodents that live their entire life underground, as well as various species of bats, live longer than other small mammals (6, 7). Extended longevity of these groups of animals is likely due to the impact of brain development and/or social organization (in the case of humans, also public health measures and medical advances) and to reduced risk of predation (for example, by living underground or flying).

Longevity also differs between members of the same species, but the range of these differences is much smaller than the differences between the species. Interestingly, within a species, it is usually the smaller, rather than the larger, individuals that live longer (8, 9). In addition to the natural variations of the lifespan due to interactions of genetic differences with environmental factors, longevity can be altered by a variety of interventions. In mammals, both average and maximal lifespan can be extended by reducing intake of food or particular nutrients (calorie restriction, dietary restriction) (10), by various drugs (11–13), and also by genetic differences due to selective breeding, spontaneous mutations, or experimentally-induced deletion or silencing of particular genes (8, 14, 15). In keeping with the reciprocal relationship of body size and longevity within the species, most of the life-extending interventions inhibit anabolic processes, growth and maturation (8, 11, 14, 16).

In this brief article, we will argue that a particular set of characteristics of the life course, the so-called “pace-of-life,” (17, 18) figures prominently among the factors responsible for both inter- and intra-specific (between and within species) differences in longevity. We will further argue that, in contrast to the relationships between body size and lifespan, slower pace-of-life is positively associated with longevity in both inter- and intra-specific (between and within species) comparisons. We will discuss relationships among growth hormone (GH), GH-dependent traits, aging, and longevity, to support both arguments.

### Phenotypic Characteristics of Mice With GH-Related Life-Extending Mutations

Genetic suppression of GH signaling leads to remarkable extension of longevity in laboratory populations of house mice (*Mus musculus*) (8, 14) (**Figure 1B**). This was shown in mice with mutations interfering with development and function of GH-producing cells in the pituitary or with hypothalamic control of GH release (3, 4, 14, 19), and in mice with targeted disruption (“knock out”) of the gene coding for GH receptor which produces complete resistance to the actions of GH (20). Severe (essentially complete) suppression of GH signals in these animals results in striking alterations of many phenotypic characteristics. These include slower postnatal growth, delayed maturation, reduced adult body size, and changes in body proportions, which together with increased adiposity, are characterized as dwarfism. In addition to the delay of puberty, dwarf mice have smaller litters and longer intervals between litters (21–23), leading to reduced fecundity (even though reproductive aging is generally delayed) (24, 25). The exact quantitative impact of dwarfing mutations on sexual maturation and fertility is strongly dependent on genetic background (8, 14), but generally resembles alterations in reproductive function in mice subjected to calorie restriction (10, 26).

Growth hormone transgenic mice, in which circulating GH levels are chronically increased above the normal range, are characterized by accelerated postnatal growth, increased adult body size, and reduced lifespan (27, 28). Interestingly, reproductive characteristics of these animals are exactly opposite to those seen in long-lived dwarf mice, and include early puberty, increased litter size, increased fecundity at a young age, as well as accelerated reproductive aging (29, 30).

Buffenstein and her colleagues (31) recently pointed out that exceptionally long-lived rodents with a slow pace-of-life are also characterized by retention of juvenile traits throughout life, the so-called “pedomorphy.” This could be interpreted as another example of trade-offs between maturation and aging.

Physiological characteristics of long-lived dwarf mice include reduced plasma levels of insulin-like growth factor-1(IGF-1, key mediator of GH actions), insulin, inflammatory markers, and lipids associated with increases in plasma adiponectin levels, insulin sensitivity, thermogenic activity of brown adipose tissue, oxygen consumption per unit body mass, and utilization of fatty acids as metabolic fuel (14, 32, 33). Most of these characteristics represent likely mechanisms of extended longevity, but may also be interpreted as markers of a younger biological age (8, 34). Both of these interpretations are generally consistent with the evidence of delayed and and/or slower aging of these mutants (8, 34, 35), and also with findings in other species and other types of long-lived mice (8, 34).

Most of the phenotypic alterations in long-lived hypopituitary Prop1^df^ (Ames dwarf) mice can be largely or completely eliminated (normalized or “rescued”) by a relatively brief period of GH replacement therapy in their early postnatal life (36). Importantly, this GH intervention also reduces longevity of Ames dwarf mice (36, 37). We interpret these findings as evidence that longevity-associated characteristics of Ames dwarf mice and their extended lifespan are mechanistically (causally) related to GH deficiency of these mutants, and that the GH signals during the period of rapid peri-pubertal growth are important determinants of adult phenotype and trajectory of aging.

Available evidence indicates that GH signaling influences aging *via* a variety of direct and indirect mechanisms. These mechanisms, along with their complex interactions, are apparently responsible for the unique “longevous” phenotypes of GH-deficient and GH-resistant individuals. Discussion of this topic is outside of the scope of the present article and an interested reader is directed to recent reviews (8, 38–40).

### Impact of Thyroid Hormones on Development and Aging

Phenotypic characteristics of animals with genetic alterations of GH signaling, discussed in the preceding section of this article, provide some extreme examples of changes in growth, development, and longevity. However, anabolic processes, maturation, and growth are regulated by a host of other factors, including nutrition and various hormones. Prominent among them are thyroid hormones (TH), which promote growth and maturation. In agreement with the concept of trade-offs between growth processes and aging, hypothyroidism has been associated with extension of longevity (41, 42), and experimentally-induced hyperthyroidism was reported to shorten life (42). Interestingly, remarkably long-lived Snell dwarf and Ames dwarf mice (Pit1 and Prop1 mutants), mentioned earlier in this article, are not only GH deficient, but also severely hypothyroid due to TSH deficiency (8, 43). The features of “pedomorphy” (retention of infantile characteristics) discussed by Buffenstein and her colleagues (31) are very prominent in these animals and have been related to hypothyroidism. Treatment of dwarf mice with TH, rapidly alters some of these characteristics (44).

In humans, hypothyroidism was reported to be associated with extreme longevity (5), and it was suggested that TH signaling at specific periods of the life course may incur costs in terms of longevity (45).

### Life Course of Long-and Short-Living Species and the Concept of Pace-of-Life

Similarly to other physical and functional characteristics, life history traits are shaped by evolution to maximize fitness under prevailing environmental conditions. For readers that may not be familiar with the concepts and terminology of evolutionary biology, we should add that we use the term “fitness” in its Darwinian sense, that is probability of producing offspring likely to be able to reproduce, rather than in its common meaning of superior physical condition and performance. Life histories and reproductive traits differ greatly between different species. Among mammals, long-lived species generally are slow to grow and reach sexual maturity, produce relatively few offspring, and invest much time and effort in their care. In contrast, short living species typically exhibit opposite traits: fast growth, early sexual maturation, and production of many offspring which quickly become independent and reproductively competent. These differences can be illustrated by comparing life history traits in house mice, a very short-lived animal, and in humans, who in comparison to other mammals are rather remarkably long-lived (**Table 1**). Differences in these and related life history traits are referred to as differences in the “pace-of-life” (17, 18). Fast pace-of-life is thought to favor current reproduction, while slow pace of life prioritizes chances for longer survival and future reproduction. This involves directing more resources to body maintenance and repair *via* a variety of molecular and cellular mechanisms. Studies of long-living species provided evidence for improved anti-oxidant defenses, increased telomerase activity, improved DNA repair and genome maintenance, as well as changes in gene expression believed to reduce risk of cancer (6–8, 11, 46, 47). Inverse relationship between pace-of-life and longevity was also shown to apply to birds and involves differences in behavioral characteristics such as readiness to undertake high risk activities (18, 48, 49). Collectively, adoption of a particular pace-of-life appears to maximize evolutionary fitness; that is reproductive success in the ecological niche occupied by the species. Differences in longevity presumably co-evolved with differences in the pace-of-life, with their reciprocal relationships resulting from complex trade-offs among processes promoting rapid growth and reproduction and traits favoring prolonged survival.

**Table 1:** Comparison of life history traits in a species with a fast pace-of-life (mouse) and a species with a slow pace-of-life (human).

	Mice	Humans
**Longevity**	~ 2 years	~ 80 years
**Total dependence on parents**	21-28 days 3-4% of lifespan	10-15 years 16% percent of lifespan
**Age at reaching reproductive competence**	40-50 days 6% of lifespan	13-16 years 18% of lifespan
**Maximal number of offspring per female**	50-100 [~10 litters with 5-10 pups per litter]	10-15

### Differences in the Pace-of-Life Contribute to Differences in Longevity Within Mammalian Species

As is described earlier in this article, mice with GH-related life-extending mutations exhibit slow growth, delayed maturation, and reduced fecundity, which are key features of the slow pace-of-life (8, 38). Mice with grossly elevated GH levels due to transgenic expression of GH genes exhibit opposite characteristics, including early puberty and large litter size (29, 30). Although pace-of-life is related to anabolic and growth processes, the relationship of pace-of-life to adult body size in these comparisons is opposite to that seen in comparisons of different species.

In this context, it is important to mention that, in mice, negative association of body size and lifespan applies not only to animals with natural or experimentally produced genetic alterations of GH signaling, but also to genetically normal (“wild type” WT) animals. This is demonstrated in the studies of mice from lines with different adult body weight (50, 51), and in tracking individual growth, body size, and longevity in mice from a genetically heterogeneous population produced by crossing four inbred strains (2). A recent study of Sandoval-Sierra and colleagues (52) evaluated epigenetic aging of female mice from well characterized lines that were originally derived from crossing relatively long-lived C57BL/6 mice with relatively short-lived DBA/2 mice. The results demonstrated that heavier body weight (a GH-dependent trait) is associated with accelerated epigenetic aging (52).

Moreover, mice derived from animals recently caught “in the wild” tend to be long-lived and to exhibit features of slow pace-of-life (late puberty and small litters) along with relatively small body size (53).

Shindyapina and her colleagues recently reported that treating mice with rapamycin during the first 45 days of their life reduced growth and adult body size, delayed puberty, promoted preservation of health, and extended median lifespan (54). This elegant study involving intervention with an anti-aging drug provides yet another example of an association of a slower pace-of-life with slower aging and indicates this relationship is almost certainly causal.

Association of slower pace-of-life with longer lifespan within a species is not limited to mice. Domestic dogs exhibit great variation of both body size and longevity. Analysis of data from individual animals or from different breeds provided strong evidence of reciprocal relationship of body size and longevity, with small dogs aging at a slower rate and living longer (9, 55, 56) Large dogs die younger and exhibit large litter size (55), an important feature of fast pace-of-life. Interestingly, metabolic rate per unit of body mass is greater in smaller than in larger dogs (57). This contrasts with reciprocal relationship of metabolic rate to longevity in comparisons between different mammalian species, but corresponds to findings in dwarf vs normal mice (32).

### Is Human Aging Related to the Pace-of-Life?

In the view of the evidence linking pace-of-life to longevity in both inter-and intra-species comparisons, it is interesting to ask whether pace-of-life also influences aging in humans. The answer to this question appears to be affirmative, although the evidence for this is largely indirect and opened to other interpretations. Earlier in this article we have already indicated that, in comparison to other mammalian species, humans are characterized by a slow pace-of-life and long lifespan. Growth hormone-related mutations that slow the pace-of-life and extend longevity in laboratory mice also have been identified in the human (38, 58, 59), and were reported to influence various aging-related traits, including risk of chronic disease. Individuals with mutations in the GH receptor gene, and the resulting GH resistance (Laron Syndrome), are almost completely protected from cancer (59, 60). Studies of a large cohort of subjects with this condition in Ecuador by Guevarra-Aguirre, Salvatori, and their colleagues, revealed almost complete protection from diabetes (59). A Brazilian population of individuals with isolated GH deficiency (IGHD) due to a mutation of the GHRH receptor gene, studied by Aguiar-Oliveira and his colleagues, exhibit numerous features of “healthy aging” including reduced risk of atherosclerosis, improved insulin sensitivity, reduced fatigue, and retention of hair pigmentation (38). In addition, subjects affected by IGHD in this cohort appear to be resistant to infections by an endemic parasite, *Leishmania* (61), and to cope better with Covid-19 infections (62). However, these characteristics appear to have little, if any, impact on their longevity (38). Information on the average lifespan of humans with these or other GH-related mutations is limited and somewhat controversial (38, 63), with the emerging consensus that these individuals have essentially normal life expectancy (38, 64). Intriguingly, a few individuals with genetic GH deficiency have reached very advanced age, including one centenarian (38, 58). Thus, evidence from studies of the individuals with genetic defects in GH signaling suggests association of a slower pace-of-life with retention of some youthful characteristics and a degree of protection from the risk of age-related disease without a clear effect on longevity.

In the general population, earlier age of puberty (a feature of accelerated pace-of-life) has been associated with negative health outcomes in adult life (65). It is interesting to speculate whether the recent trend for nutritionally-driven acceleration of sexual maturation may play a role in the stalling and/or likely reversal of historical trends for progressive increase in longevity (66, 67). Discussion of the nutritional, public health, medical and socio-economic factors likely to have impacts on these trends is outside the scope of this brief article. Identification of mechanisms underpinning the relationships between pace of life and longevity will help in designing practical interventions to promote healthy aging.

## Author Contributions

The author confirms being the sole contributor of this work and has approved it for publication.

## Funding

Writing of this article and our recent and current studies of this topic were supported by grant NIA R21-AG062985.

## Conflict of Interest

The author declares that the research was conducted in the absence of any commercial or financial relationships that could be construed as a potential conflict of interest.

## Publisher’s Note

All claims expressed in this article are solely those of the authors and do not necessarily represent those of their affiliated organizations, or those of the publisher, the editors and the reviewers. Any product that may be evaluated in this article, or claim that may be made by its manufacturer, is not guaranteed or endorsed by the publisher.
